# Oxidative C−C bond cleavage of lignin *via* electrocatalysis

**DOI:** 10.3389/fchem.2022.1007707

**Published:** 2022-09-15

**Authors:** Jinshu Huang, Yumei Jian, Min Zhou, Hongguo Wu

**Affiliations:** ^1^ State Key Laboratory Breeding Base of Green Pesticide and Agricultural Bioengineering, Key Laboratory of Green Pesticide and Agricultural Bioengineering, Ministry of Education, State-Local Joint Laboratory for Comprehensive Utilization of Biomass, Center for R&D of Fine Chemicals, Guizhou University, Guiyang, China; ^2^ College of Materials Science and Engineering, Guizhou Minzu University, Guiyang, China

**Keywords:** biomass conversion, lignin, biofuels, electrocatalysis, green chemistry

## Abstract

Lignin, which is an important component of biomass in nature and is constantly produced in industry, becomes potential raw material for sustainable production of fine chemicals and biofuels. Electrocatalysis has been extensively involved in the activation of simple molecules and cleavage-recasting of complex scaffolds in an elegant environment. As such, electrocatalytic cleavage of C−C(O) in *β*-O-4 model molecules of lignin to value-added chemicals has received much attention in recent years. This mini-review introduces various anodes (e.g., Pb, Pt, Ni, Co., and Ir) developed for electro-oxidative lignin degradation (EOLD) under mild conditions. Attention was placed to understand the conversion pathways and involved reaction mechanisms during EOLD, with emphasis on the product distribution caused by different electrodes.

## Introduction

**FIGURE 1:**
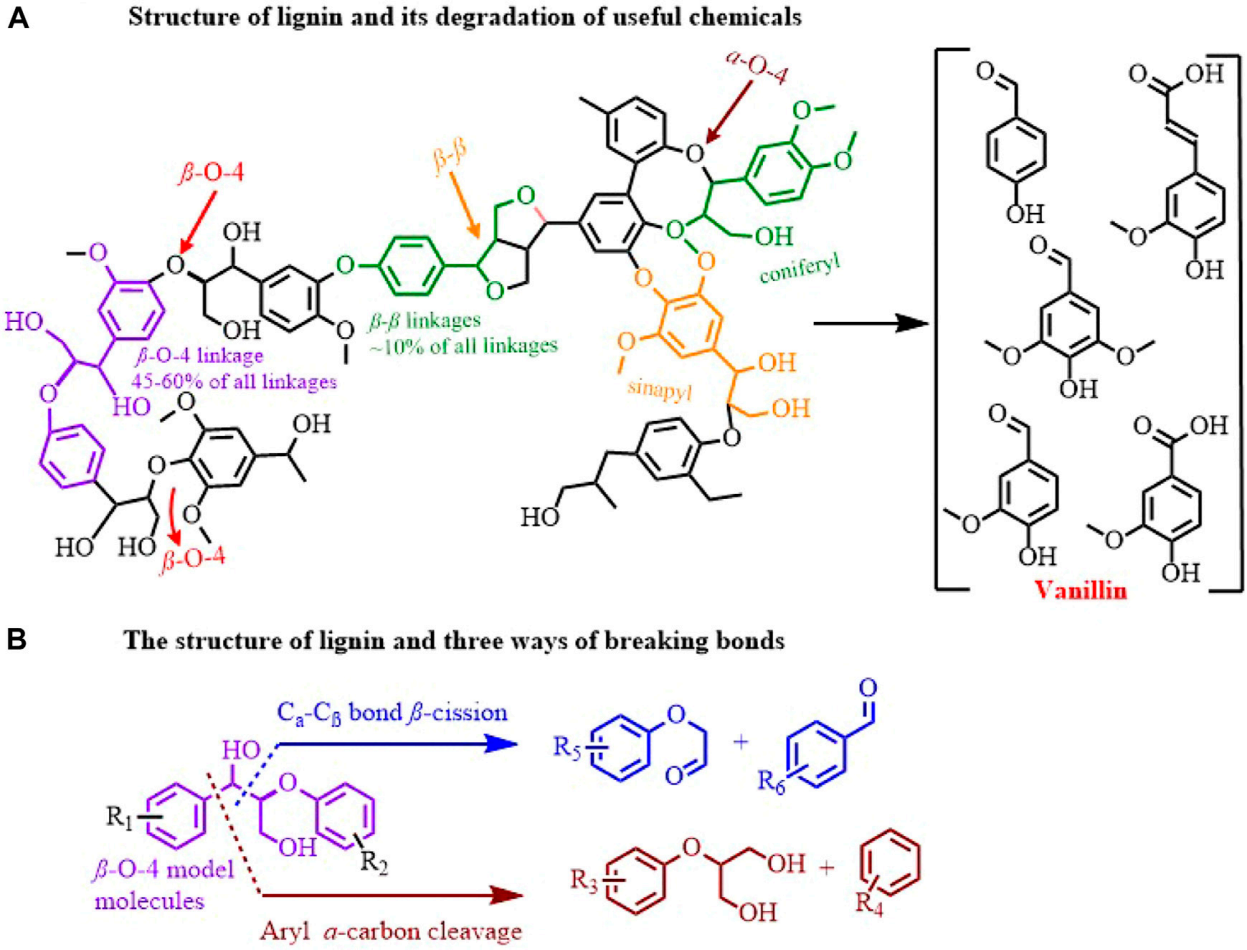
Schematic of the structure and bond cleavage position **(A)**, and fracture mode **(B)** of lignin..

Lignin, which is a large amount of biomass in nature and is constantly produced in industry, becomes potential raw material for sustainable production of fine chemicals, biofuels and functional materials, considering its polyphenolic structure and carbon-rich properties (Pardini et al., 2002; Li et al., 2019; Wu et al., 2021; Huang et al., 2022; Jian et al., 2022). Especially, catalytic cleavage of C−C(O) in *β*- O-4 model molecules has received much attention (Sun et al., 2018; Yu et al., 2022). Traditional *β*- O-4 model molecule degradation methods, including pyrolysis, catalytic hydrodeoxygenation, liquefaction, and oxidative cracking (Jia et al., 2018; Valle et al., 2013), can efficiently convert lignin into value-added fine chemicals (Figure 1A). Definitely, those methods have many areas to be improved, such as harsh conditions and non-specific selectivity. Alternatively, electro-oxidative lignin degradation (EOLD) is a mild and sustainable method that featured selective cleavage of the C−C bond (C_*α*_−C_*β*_ bond and *β*- O-4 ether cleavage) using user-friendly electron while retaining the inherent aromatic structure (Figure 1B) (Liu et al., 2019; Di Fidio et al., 2021).

In 1946, Bailey and Brooks firstly illustrated that the metal materials (e.g., Pb, Cd, Pt, and Ag) serving as anodes for the electrocatalytic oxidation of alkali lignin or methylated butanol lignin could successfully furnish methyl ethyl ketone, acetone, and acetic acid (Figueiredo et al., 2017). Since then, a growing number of researchers utilized metals such as Pb, Pt and Ni as anodes to conduct EOLD. The following sections are a detailed introduction to the classification of these anodic materials.

## Lead/lead oxide electrodes

Lead/lead oxide electrodes are extensively used as anode materials for EOLD due to their superior chemical stability in aggressive media, high overpotentials in oxygen evolution reactions (competitive reactions), and low prices (Quiroz et al., 2005). In the presence of (+) (Pb/PbO_2)_/SS (−) with an increased specific surface area, 4-methylanisole was mainly generated by the electrooxidation of lignin through C_*α*_−C_*β*_ bond breaking with a free radical (·OH). When the current density was 50 mA/cm^2^ at 50°C, the yield of product 4-methylanisole is the highest (Wang et al., 2015). It is worth noting that a high temperature will lead to the inactivation of the catalytic reaction free radical. In the same device, the cyclic voltammetry and cathodic polarization indicated that the copper electrode could reduce the hydrogenation rate and electrocatalytic hydrogenation rate of the hydrogen atom electrochemical solution (Liu et al., 2017). The yield of syringaldehyde was 57.30 g (kg-lignin)^−1^, which is higher than that of vanillin and *p*-coumaric acid at a lower current density (20 mA/cm^2^) and temperature (40°C). The same device could also be used to oxidize alkaline lignin to butyl hydroxytoluene (BHT) (Zhang et al., 2014). For the degradation process, electrochemical impedance spectroscopy and cyclic voltammetry show that the phenolic hydroxyl monomolecular structure in lignin is combined with sodium ions in the electrolyte to form sodium phenolate, and then the aromatic ring of the C−C bond was highly induced and selectively cleaved with superoxide anion radical (O_2_^•−^) on the anodic surface of Pb/PbO_2_, thus generating lignin superoxide anion radical. Finally, the branched chain of the benzene is cleaved, and then the intermediate is deoxygenated by cathodic protons. It was further converted into BHT by the electrophilic attack of *tert*-butyl carbophenyl ammonium ion with a final separation yield of 7.01% under constant current conditions (25 mA/cm^2^), and the concentration of BHT was negatively correlated with the current density. When using Ti/PbO_2_ and Ti/Sb-SnO_2_ as electrocatalysts in the pre-degradation of sodium lignosulfonate solution (2000 ppm) (Shao et al., 2014), quinone and short-chain carboxylic acid are essential intermediates and primary products, respectively. The results showed that Ti/Sb-SnO_2_ and Ti/PbO_2_ had different advantages in their UV-visible absorption reduction, chemical oxygen demand (COD) removal capacity, and biodegradability. In the same device, the Ti/SnO_2_-Sb_2_O_3_/*α*-Pb O _2_/*β*-Pb O _2_ electrode was prepared by further modification of Ti/Sb-SnO_2_ and Ti/PbO_2_ electrodes, and Ir or Ti-doped Ti/Cu/Sn electrode as a cathode. Interestingly, the products could be oriented toward aromatic ketone, aldehyde, or acid when Ti/Cu/Sn was used as the cathodic material, proving that cathodic materials with different titanium-based materials had important effects on the process and products of EOLD.

The lead oxide coating prepared by the conventional method is easy to peel off from the surface of the substrate owing to its relatively high interfacial resistance (Hao et al., 2015). This problem can be improved by photoelectrical deposition of lead oxide onto TiO_2_ nanotubes (NTs) arrays. The NTs can increase the available surface area of the electrode, thus improving the load capacity of lead oxide (Pan et al., 2012). The prepared Ti/TiO_2_NT/PbO_2_ electrode showed a high electrochemical response and lasting stability, which was active for the crack of the C_*α*_−C_*β*_ bonds of kraft lignin to give vanillin and vanillin acid. The [Fe(CN)_6_]^3−^ modified lead oxide electrode prepared by deposition method has a wider central active surface area, resulting in the formation of more OH radicals and their adsorption sites, closely correlated with significantly increased active sites (Hao et al., 2015). It is worth noting that implantation of [Fe(CN)_6_]^3−^ anion into the lead oxide matrix is beneficial to the growth of lead oxide crystals, thus optimizing the size and load capacity of lead oxide electrodes. Overall, the [Fe(CN)_6_]^3−^-modified lead oxide electrode can effectively reduce interfacial resistance and thus effectively prevent stripping. The accelerated life test data showed that the life of the modified PbO_2_ electrode was 0.8 times longer than that of the bare PbO_2_ electrode, and the degradation rate constant significantly increased from 0.00419 to 0.00609 min^−1^, but the product category is not specified in this literature.

## Platinum electrodes

Pt was usually developed as a hydrolysis catalyst and anodic electrode material to catalyze the electrically oxidative fracture of the C_*α*_−C_*β*_ bond in the degradation and utilization of lignin (Liu et al., 2019). One-pot degradation of lignin by using hydrogen peroxide *tert*-butyl (*t*-BuOOH, 70%) as an oxidant, Pt as electrode material, where C_*α*_−C_*β*_ bonds were cleaved specifically into functional aromatic hydrocarbons (e.g., 3-methoxy-benzaldehyde in 81% yield and phenol in 43% yield) (Ma et al., 2021). Mechanism studies show that the reaction pathway undergoes through forming *in situ* C_*β*_-centered free radicals to produce peroxide intermediates and further inducing oxidative cleavage of C_*α*_−C_*β*_ bond to give 3-methoxy-benzaldehyde. For highly dispersed single-atom Pt−N_3_C_1_ nanotubes, the increase in single-atom unsaturated coordination number results in the increase of the active sites, while the high degree of dispersion can improve the atomic utilization, thereby increasing the activity and yield per unit catalyst. The results of electrical experiments show that Pt_1_/N-CNTs have high selectivity and activity for activating C_*α*_−C_*β*_ bonds in lignin. Density functional theory (DFT) calculation proves that the C-center free-radical intermediate is formed in the degradation process, and the unstable C_*β*_-radical undergoes a cross-coupling reaction to generate the peroxide intermediate. After the reaction, electron transfer results in C_*α*_−C_*β*_ bond-breaking to provide benzaldehyde (81% yield) (Cui et al., 2021).

## Nickel−, cobalt−, and nickel–cobalt−based electrodes

Ni-electrode has excellent chemical resistance and is widely used in EOLD. Ni is used as an anode and flow reactor (FM01) device to prepare vanillin (Masoumi et al., 2016). Control experiments showed that optimizing current density and adjusting the initial concentration of lignosulfonate can obviously improve the yield of vanillin, in which Ni as anode has two competitive reactions in lignin degradation. The reaction is oxidized by nickel (II) to the oxygen-containing nickel (III) species (Ni(III)OOH). A maximum vanillin yield of 7.4% w/w could be obtained from 1.5% w/v lignin at 130°C and 1.9 mA/cm^2^. Unsatisfactorily, the complexity of the experimental device and the conditions of high temperature and high pressure hinder the development of this method. Instead, changing the simple device can make the reaction easy to operate, and the new device “Swiss coil” electrochemical reactor and Ni (foam) electrode using water as an electrolyte are applied to EOLD (Di Marino et al., 2019). Starting from lignin, the C−C bond is initially destroyed to form a carboxylic acid compound and then broken into a low molecular compound, such as vanillin. Free radicals can be reduced and generated by oxygen transfer from the anode to the cathode, which can promote the decrease of the average molecular weight of lignin to obtain value-added products. Di- and mono-carboxylic acids (e.g., ethanedioic acid, vinegar acid, and formic acid) have high reactivity, with the formic acid yield of 26.8%, which was higher than oxalic acid and acetic acid.

In the unstable electrochemical depolymerization process, a dynamic model was constructed to predict the formula weight change of the EOLD in the reaction device, and the simulation of the reaction (chain break, random recombination, and random fracture) is high similarity (Bawareth et al., 2018). After introducing a contributor to the overall reaction, the model predicts the function of lignin degradation and product formation simultaneously, and the effect of the three major reactions is similar to the experimental data. It is further indicated that the reaction rate coefficient is linearly related to the initial multispecies and mean molecular weight of lignin. Also, a membrane reactor was applied to EOLD. The obtained results showed that when the membrane pore size was 1 nm, the aromatic hydrocarbon yield in the batch reactor could be increased from 0.01% to 11% (Bawareth et al., 2018). In general, the Ni-electrode is stable and does not fall off, but the activity is not ideal, which limits the application range of the Ni-based electrodes.

Cobalt oxide (CoO_x_) electrodes show outstanding activity in EOLD and can significantly improve the yield of vanillin, but are easily detached from the electrolyte to lose the function of transferring electrons, which blocks its industrial applications. The stable Co. core/Pt shell structure is not only conducive to electron transport, but also effectively avoids the problem of easy shedding of exposed Co., and it was successfully prepared by the polyol method and applied for EOLD (Movil-Cabrera et al., 2016). The main products are heptane and apocynin, although the type and yield of the product vary with the electrode potential. Other oxidative products (e.g., 1,3-bis(1,1-dimethylethyl)-benzene and 1,4-di-*tert*-butyl phenol) may participate in the oxidative decomposition initiated by free radicals in an alkaline medium.

It has been shown that a variety of metal doping and multi-metal alloy electrode materials can not only avoid the inherent defects of the single metal itself, but also show the advantages of their respective metals (Cai et al., 2014). For example, Ni-Co co-based materials exhibited outstanding activity and excellent corrosion resistance. Waldvogel (Schmitt et al., 2015) and Zirbesl **(** Cai et al., 2014 **)** both added a Co-based anode to a Ni-based anode for EOLD to improve the yield of vanillin. The former forms an electrochemically active NiOOH coating *in situ*, and the electrolysis conditions are optimized when the reaction temperature is lower than 100°C (Schmitt et al., 2015). The use of strongly basic anion exchange resin can selectively remove the low molecular weight of phenol in the strongly basic electrolyte, so it is unnecessary to acidify and precipitate the remaining lignin. Dissimilarly, Zirbes et al. (2019) electrochemically activated the electrode in black liquor, which was demonstrated to significantly increase the electrocatalytic activity and the Ni(foam)-electrode could be reused 6-times (Garedew et al., 2020). It was found that diaminotoluene was the main product in the adsorption layer, indicating that the compound was involved in the activation process to a certain extent. Else, the deposited organic surface layer can not only increase the lipophilicity of the electrode surface but also further promote the adsorption and oxidative degradation of lignin, thus obtaining a good yield (0.9 wt%) of vanillin. The mechanism of both sets of experiments was that in an alkaline electrolyte, an electrocatalytically active NiOOH layer was formed on the surface of the anode Ni (Schutyser et al., 2018), in which EOLD enhanced the oxidative activity of the electrode, while helping to avoid further peroxidation of the formed monomers (Smith et al., 2010), and thereby significantly increasing yield. Different from platinum and other expensive metal or large pieces of the metal electrode (e.g., large, and flat electrode), nanoparticle catalysts potentially show increased activity in electrically catalytic degradation of lignin, owing to a higher utilization rate of metal, which can promote the quality of the reactants and products through optimizing the structure of electrode materials.

## Iridium oxide electrodes

The unexpected electrocatalytic selectivity and corrosion resistance of iridium oxide electrodes have attracted the wide attention of investigators (Trasatti, 2000). Different IrO_2_-based electrodes (e.g., Ti/MO-IrO_2_, MO = SnO_2_, RuO_2_, Ta_2_O_5_, and TiO_2_) were prepared and applied to the EOLD (Tolba et al., 2010). The cyclic voltammetry curves show that the electrochemically active surface areas of the four metal/oxide species modified IrO_2_ electrodes exhibit the following sequence: Ti/Ta_2_O_5_-IrO_2_ > Ti/TiO_2_-IrO_2_ > Ti/SnO_2_-IrO_2_ > Ti/RuO_2_-IrO_2_. The good stability and highest reaction rate constant (apparent activation energy of electrochemical oxidation: 20 kJ/mol) indicate that the resulting hydroxyl radicals are advantageous for the break of the C−C bond in lignin. At 60°C, the optimum current density was 500 mA cm^−2^, and the yield of vanilla and vanillic acid reached the maximum value (500 ppm lignin). A binary mixed metal oxide (MMO) (Ru_0.4_Ir_0.6_O_x_) electrode derived from ruthenium and osmium oxide was successfully prepared and found to show good activity in electrocatalytic degradation of lignin to produce diaphylin (Zhu et al., 2014), but the decomposition of electrolyte causes electrochemical windows very narrow. Also, by using transition metals to modify the binary Ru_0.4_Ir_0.6_O_x_-electrode for the preparation of three-membered MMO electrodes (Ru_0.2_M_0.2_Ir_0.6_O_x_; M = Mn, Pd, V, and Ti), the activity of Ru_0.2_M_0.2_Ir_0.6_O_x_-electrodes is higher than binary Ru_0.4_Ir_0.6_O_x_-electrode, for example, the Ru_0.2_Mn_0.2_Ir_0.6_O_x_ has the highest electrocatalytic activity (11.5% yield) (Rauber et al., 2018). Moreover, the composition of the electrode also changes the number of cracking products and the selectivity of the break button. The reaction was carried out in ionic liquids ([Et_3_NH][MeSO_3_]) considering that [Et_3_NH][MeSO_3_] has quantitative turnover and no side reaction (Achinivu et al., 2014), as well as does not produce any contaminant, and shows high electrochemical windows through some synergy, such as the hydrophilicity of ILs and the hydrophobicity of the aromatic pyrolysis products allow the product to be separated from the untreated lignin. Lignin can be oxidized directly at the anode or the cathode (Garedew et al., 2020). In a nondiaphragm cylindrical electrolytic cell, the graphite cathode is on the inside, and the RuO_2_-IrO_2_/Ti net anode is depolymerized in an alkaline aqueous solution on the outside. The by-product O_2_ on the anode can be effectively reduced to H_2_O_2_ on the cathode. Also, ·OH, ·O_2_^−^ and OOH are decomposed into H_2_O_2_ (Moodley et al., 2012). As such, lignin is broken by these free radicals and the C−C bonds are anodized to produce aromatic products in different low molecular weights.

Lignin can directly form aromatic compounds by breaking the C−C bond. Some types of lignin can be oxidized into intermediates such as acids and ketones first, followed by decomposition of the C−C bond to generate vanillin (Schutyser et al., 2018), benzaldehyde, and other products. This class of lignin typically contains two hydroxyl groups at the *β-* O-4 position (Bosque et al., 2017), benzyl alcohol on C_*α*_ and aliphatic alcohol on C_*γ*_. Such structures have a high degree of specificity of electron receptors, making the structure prone to highly selective rupture of the C_*α*_−C_*β*_ bond (Karkas et al., 2016). Overall, the single alcohol in *β*- O-4 lignin can undergo highly selective oxidation to furnish oxidative intermediates, and these intermediates could proceed through C−C and/or C−O bonds cleavage to obtain single aromatic products.

## Conclusion

In summary, a variety of electrode materials prepared with different advantages (e.g., high activity, good stability, easy availability, and high selectivity to C−C bonds break, and electrodes with multiple metals) are demonstrated to show unexpected comprehensive effects. Different reaction devices are also illustrated to have an impact on the product distribution and yield in the electrocatalytic degradation of lignin. The reaction mechanisms involve the generation of free radicals (e.g., O_2_·^−^, andOH), and the formation of MOOH to induce the C−C bond breakage. The combination and development of the already well-established electrocatalytic cleavage technology and the much-touted biomass conversion are desired to usher another industrial renaissance in the domain of chemical synthesis.
